# Serine protease inhibitors decrease metastasis in prostate, breast, and ovarian cancers

**DOI:** 10.1002/1878-0261.13513

**Published:** 2023-09-25

**Authors:** Amiram Sananes, Itay Cohen, Irit Allon, Oshrit Ben‐David, Raghda Abu Shareb, Ksenia M. Yegodayev, David Stepensky, Moshe Elkabets, Niv Papo

**Affiliations:** ^1^ Avram and Stella Goldstein‐Goren Department of Biotechnology Engineering and the National Institute of Biotechnology in the Negev Ben‐Gurion University of the Negev Beer‐Sheva Israel; ^2^ Institute of Pathology, Barzilai University Medical Center, Ashkelon, Israel ad Faculty of Health Sciences Ben‐Gurion University of the Negev Beer‐Sheva Israel; ^3^ The Shraga Segal Department of Microbiology, Immunology, and Genetics, Faculty of Health Sciences Ben‐Gurion University of the Negev Beer‐Sheva Israel; ^4^ Department of Clinical Biochemistry and Pharmacology, Faculty of Health Sciences Ben‐Gurion University of the Negev Beer‐Sheva Israel

**Keywords:** cancer imaging, kallikreins, metastasis, protease inhibitors, serine proteases

## Abstract

Targeted therapies for prostate, breast, and ovarian cancers are based on their activity against primary tumors rather than their anti‐metastatic activity. Consequently, there is an urgent need for new agents targeting the metastatic process. Emerging evidence correlates *in vitro* and *in vivo* cancer invasion and metastasis with increased activity of the proteases mesotrypsin (prostate and breast cancer) and kallikrein 6 (KLK6; ovarian cancer). Thus, mesotrypsin and KLK6 are attractive putative targets for therapeutic intervention. As potential therapeutics for advanced metastatic prostate, breast, and ovarian cancers, we report novel mesotrypsin‐ and KLK6‐based therapies, based on our previously developed mutants of the human amyloid β‐protein precursor Kunitz protease inhibitor domain (APPI). These mutants, designated APPI‐3M (prostate and breast cancer) and APPI‐4M (ovarian cancer), demonstrated significant accumulation in tumors and therapeutic efficacy in orthotopic preclinical models, with the advantages of long retention times *in vivo*, high affinity and favorable pharmacokinetic properties. The applicability of the APPIs, as a novel therapy and for imaging purposes, is supported by their good safety profile and their controlled and scalable manufacturability in bioreactors.

## Graphical abstract

As a means to address the urgent need for new agents targeting the metastatic process, this study focuses on the optimization, characterization, and testing of novel mesotrypsin‐ and KLK6‐based therapies. We focus on prostate, breast, and ovarian cancers as proof of concept, but these agents can potentially also be used for other types of cancer.

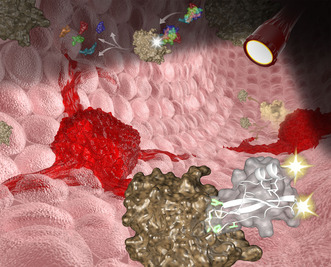

**Abbreviations**
APPI: human amyloid β‐protein precursor Kunitz protease inhibitor domain
BGU: Ben‐Gurion University of the Negev
EMT: epithelial‐to‐mesenchymal transition
FBS: fetal bovine serum
HSA: human serum albumin
KLK: Human tissue kallikrein
PSA: prostate‐specific antigen
SCID: severe combined immunodeficient
TME: tumor microenvironment

## 1 Introduction

Cancer is a major cause of morbidity and mortality, with prostate and breast cancers being the most commonly diagnosed cancers in men and women, respectively [1]. While rarer in incidence than prostate cancer, ovarian cancer is the most lethal gynecological malignancy, accounting for 5–6% of all cancer‐related deaths in women [1, 2]. For the above cancers, metastasis – the spread of cancer cells from the original tumor to other organs – is the leading cause of death [3, 4, 5]. Thus, the development of new therapies to combat metastasis is clearly of core importance in winning the war against these, and other, types of cancer.

Significant contributors to the development of metastases are extracellular proteases, which are usually overexpressed in the tumor microenvironment (TME) [6, 7]. Noteworthy among these proteases is mesotrypsin (also known as PRSS3), a serine endopeptidase belonging to the chymotrypsin superfamily and associated with increased malignancy in several cancers, including lung, pancreas, breast, and prostate cancers [8, 9, 10, 11]. It has been shown, for example, that mesotrypsin is upregulated in T4‐2 breast cancer cells and that knockdown of mesotrypsin attenuates the malignant phenotype of the cells, while treatment with recombinant purified mesotrypsin enhances it [10].

Mesotrypsin upregulation has also been associated with metastatic prostate tumors [12]. It has been shown that its increase in the primary tumor is prognostic for a high recurrence of cancer following prostatectomy [9]. Conversely, silencing mesotrypsin reduced prostate cancer cell sprouting in culture. Furthermore, using a murine model of prostate cancer cells transplanted into the prostate (orthotopic model), the Radisky group showed that primary tumor progression and metastasis formation were markedly decreased in mice with transplanted mesotrypsin‐silenced cancerous cells vs. mice with transplanted non‐silenced cancerous cells [9]. In addition, injection of recombinant mesotrypsin promoted an invasive phenotype of the cancerous cells. The specificity of the proteolytic activity was supported by the fact that neither cationic trypsin nor non‐catalytic mesotrypsin drove the invasive phenotype [9].

Despite their potential for anti‐metastasis therapy, only a limited number of potent mesotrypsin inhibitors have been described to date [13, 14, 15, 16, 17, 18, 19]. This knowledge lacuna may relate to the fact that mesotrypsin is structurally related to similar proteases, thereby making specific targeting a challenging endeavor [20]. Furthermore, mesotrypsin is resistant to inhibition by serine protease inhibitors [21, 22, 23, 24]. Despite these drawbacks, the recent discovery (by X‐ray crystallography) of mesotrypsin‐specific active sites has enabled us to select proteolytically resistant inhibitors by using rational and combinatorial approaches [25, 26].

Another serine endopeptidase family implicated in malignancy comprises the human kallikrein‐related peptidases, KLKs. Some KLKs are therefore used as tumor biomarkers; for example, KLK3 [prostate‐specific antigen (PSA)] serves as a biomarker for prostate cancer [27]. Recently, KLKs have been described as critical regulatory proteases that are tissue specific [28]. In this context, KLK6 is significantly upregulated in ovarian cancer [29] (in addition to other types of cancer [30, 31]). An investigation of its role in ovarian cancer invasion revealed its involvement in cell signaling, the development of metastases, and poor survival [32]. It has also been shown that KLK6 triggers tumor cells and parenchymal cells in the TME and that the metastasis‐promoting activity of KLK6 may be mediated by its ability to degrade extracellular matrix proteins and E‐cadherin in parallel to the epithelial‐to‐mesenchymal transition (EMT) [33]. In addition, it has been shown that RNA‐interference‐suppressed KLK6 impedes tumor cell growth and the formation of metastases, while ectopic KLK6 expression increases ovarian tumor proliferation [34, 35].

In any attempt to develop inhibitors for serine endopeptidases of the KLK family [36, 37, 38] or the trypsin‐like protease family [9, 21], such as KLK6 or mesotrypsin, the issue of drug selectivity is critical, as these structurally and functionally similar enzymes perform a variety of essential biological functions and/or they may have anticancer activity [39]. For example, these families include many enzymes with crucial functions in blood coagulation, fibrinolysis, the complement pathway, digestion, reproduction, and wound healing [40, 41]. In the work that has been performed to date on developing specific inhibitors, a recent report described depsipeptide KLK inhibitors with enhanced activity against KLK6, but most studies have not reported activity against related proteases [42]. Nonetheless, some studies have investigated a protein‐based agent targeting mesotrypsin and KLK6, i.e., the human amyloid β‐protein precursor Kunitz protease inhibitor domain (APPI), a member of the human Kunitz domain family of serine protease inhibitors [43]. Although this agent is a broad‐spectrum, non‐selective inhibitor of trypsin‐like serine proteases, it has attracted our interest as a scaffold for engineering tumor‐targeting proteins because it is a small, compact molecule (58 amino acids) stabilized by a hydrophobic core and three disulfide bonds, resulting in high thermal stability [43].

In a previous study, we identified a triple mutant, designated APPI_M17G/I18F/F34V_ (or, in short, APPI‐3M), with a mesotrypsin inhibition constant (*K*_*i*_) of 89 pm, as the most potent mesotrypsin inhibitor yet reported. This variant is characterized by a 1459‐fold improved affinity to mesotrypsin vs. wild‐type APPI (APPI_WT_), up to 350 000‐fold greater specificity to mesotrypsin vs. other tested human serine proteases (i.e., KLK6, anionic trypsin, cationic trypsin and factor XIa), and 83‐fold improved proteolytic stability vs. APPI_WT_. We demonstrated that APPI‐3M acts as a functional inhibitor in cell‐based models of mesotrypsin‐dependent prostate cancer cellular invasiveness [25]. We also generated a quadruple variant, APPI_M17L,I18F,S19F,F34V_ (APPI‐4M), an APPI variant with a KLK6 inhibition constant (*K*_*i*_) of 160 pm and a turnover time of 10 days. To the best of our knowledge, APPI‐4M is the most potent protein‐based KLK6 inhibitor reported to date, displaying 146‐fold improved affinity, up to 40‐fold weaker inhibition potency toward other serine proteases and KLK family members, and 13‐fold improved proteolytic stability compared with wild‐type APPI_WT_. We further demonstrated that APPI‐4M is a functional inhibitor in a cell‐based model of KLK6‐dependent breast cancer invasion [44].

Here, we report, for the first time, mesotrypsin‐ and KLK6‐targeted therapies derived from human APPI for advanced metastatic prostate, breast, and ovarian cancers. APPI‐3M accumulates in prostate and breast cancer tumors and metastatic sites, and APPI‐4M concentrates in ovarian cancer sites. When fused to human serum albumin (HSA), both inhibitors exhibit a long retention time *in vivo*, high affinity for their targets, favorable pharmacokinetic properties, and significant therapeutic efficacy, as demonstrated in orthotopic preclinical models. The utility of the APPI inhibitors as a novel therapy and for imaging purposes is supported by a good safety profile and scalable manufacturability in bioreactors.

## 2 Materials and methods

### 2.1 Ethics statement

This study and protocols were approved by the Ben‐Gurion University Committee for the Ethical Care and Use of Animals in Experiments (permit number 95.11.19). All surgeries were performed under anesthesia, and efforts were made to minimize discomfort, distress, and suffering.

### 2.2 Production of human kallikrein 6

Recombinant pro‐KLK6 (with the stabilizing mutations R74G, R76Q, and N132Q [36]) was expressed and purified from the virus/insect cell line system Sf21 at the Protein Expression and Purification Core Facility, EMBL Heidelberg, Germany, as described previously [45]. The pro‐enzyme was activated with enterokinase. The mature enzyme was purified, and its concentration was estimated by UV–visible absorbance at 280 nm with an extinction coefficient (ε_280_) of 34.67 × 10^3^ m ^−1^ × cm^−1^ in a DeNovix DS‐11 spectrophotometer (DeNovix, Wilmington, DE, USA).

### 2.3 PEGylation of APPI‐3M and APPI‐4M

APPI‐3M and APPI‐4M were PEGylated with the amine‐reactive methoxy PEG succinimidyl carboxymethyl ester (Jenkem, Plano, TX, USA) of molecular weight 20 kDa. For PEGylation, 920 μg of APPI‐3M or APPI‐4M were mounted on 100 μL of nickel‐nitrotriacetic acid (NTA)‐Sepharose beads (Invitrogen, Waltham, MA, USA) that bind to the His‐tag at the C‐terminus of the APPI proteins in a binding buffer (PBS containing 10 mm imidazole). The reactants were mixed in a rotisserie incubator for 10 min at room temperature. The beads were sedimented using a table‐top centrifuge for 1 min at 2348 ***g***. The supernatant was discarded, and the beads were washed with 200 μL of 0.1 m sodium bicarbonate, pH 8.3. After a second step of washing with binding buffer to remove any unbound APPI‐3M or APPI‐4M from the PEGylation reaction, the beads were resuspended in 200 μL of 0.1 m sodium bicarbonate, pH 8.3. APPI‐3M‐ or APPI‐4M‐bound beads were then transferred to a new tube containing 200 mg PEG and centrifuged at 6010 ***g*** for 2 min to solubilize the PEG. The PEGylation reaction mixture was incubated at 25 °C for 24 h. The mixture was washed three times with binding buffer, with centrifugation steps in between, to remove any non‐reacted PEG. Three steps of protein elution were performed using 500 μL of elution buffer (PBS containing 300 mm imidazole). The eluted PEGylated protein fractions were separated using a Superdex 75 16/600 column (GE Healthcare, Westborough, MA, USA). Fraction samples were analyzed by SDS/PAGE using 15% polyacrylamide under reducing conditions.

### 2.4 Production of APPI‐3M‐HSA and APPI‐4M‐HSA fusion proteins

HSA was fused via the short GGGGS flexible linker to the C‐terminal side of APPI‐3M or APPI‐4M [25, 44]. Cloning was performed by GeneScript (Piscataway, NJ, USA) using the pPICK9K plasmid (Sigma‐Aldrich, St. Louis, MO, USA). To insert APPI‐3M or APPI‐4M genes into the HSA‐containing plasmid, both the gene and the HSA‐containing vector were digested with EcoRI and AvrII restriction enzymes (New England Biolabs, Ipswich, MA, USA), and the digested products were purified from an agarose gel. The digested fragments were then ligated, and the ligation products were transferred into DH10B‐competent bacteria. pPICK9K comprising APPI‐3M‐HSA or APPI‐4M‐HSA was then purified using MaxiPrep (Machery‐Nagel, Dueren, Germany) and transformed into GS115 *Pichia pastoris* yeast (Sigma‐Aldrich) according to the manufacturer's protocol. PEGylated APPI and HSA‐APPI fusions were evaluated for their ability to inhibit KLK6, and their *K*_*i*_ values were determined as previously described [44]. Experiments were performed in triplicate, and SD values were determined.

### 2.5 Large‐scale production of APPI and APPI fusion proteins

APPI‐3M, APPI‐4M, APPI‐3M‐HSA, or APPI‐4M‐HSA were expressed in a Jupiter bioreactor (Solaris Biotechnology, Porto Mantovano, Italy), as follows. *P. pastoris* GS115‐APPI clones [25] were first inoculated into 50 mL of BMGY medium (1% yeast extract, 2% peptone, 0.23% potassium phosphate monobasic, 1.18% potassium phosphate dibasic, 1.34% yeast nitrogen base, 4 × 10^−5^% biotin, and 1% glycerol) to an A_600_ = 10.0 (10^8^ cells·mL^−1^), followed by scaling‐up to 500 mL of BMGY until an A_600_ = 10.0 was reached (overnight growth at 30 °C with shaking at 300 r.p.m.). Thereafter, the overnight culture of yeast was added to 5 L of FM22 medium [315 mm KH_2_PO_4_, 38 mm (NH_4_)_2_ SO_4_, 6 mm CaSO_4_, 82 mm K_2_SO_4_, 240 mm MgSO_4_, 4% glycerol, pH = 5.0] and 50 mL of PTM (8 mm CuSO_4_, 0.5 mm NaI, 17 mm MnSO_4_, 0.8 mm Na_2_MoO_4_, 0.3 mm H_3_BO_3_, 3 mm CaSO_4_, 4 mm CoCl_2_, 51 mm ZnCl_2_, 144 mm FeSO_4_, 0.8 mm biotin, and 0.1% H_2_SO_4_). Cells were grown with stirring under 30% dO_2_ (delivered oxygen). When the glycerol in the medium had been completely consumed by the yeast cells, seen as an increase in soluble O_2_, MeOH was added in a concentration that was adjusted according to wet cell mass as previously described [25]. After 3 days of fermentation, the medium was collected and centrifuged at 4279 ***g*** for 20 min. Thereafter, a solution of 300 mm NaCl and 10 mm imidazole was added to the supernatant, the pH was adjusted to 8.0, and the mixture was incubated for 1 h at 4 °C. After centrifugation and filtration through a 0.22‐μm filter (PALL, Port Washington, NY, USA), the filtered medium was loaded on HisTrap™ nickel columns (GE Healthcare). The columns were washed with wash buffer (PBS, 20 mm imidazole, pH 8.0) and eluted with elution buffer (PBS, 500 mm imidazole, pH 8.0) on an ÄKTA FPLC™ Fast Protein Liquid Chromatograph (GE Healthcare). The eluted protein was separated in PBS using a Superdex 75 16/600 column (GE Healthcare).

### 2.6 Cell cultures

PC3‐M (RRID:CVCL_0035) Red‐FLuc cells (luciferase‐expressing PC3‐M cell line) were obtained from PerkinElmer (Norwalk, CT, USA) and maintained in RPMI‐1640 medium supplemented with 10% FBS. OVCAR‐3 (RRID:CVCL_0465) LUC cells (luciferase‐expressing OVCAR‐3 cell line) were obtained from JCRB Cell Bank (Osaka, Japan) and were maintained in RPMI‐1640 medium with 0.01 mg·mL^−1^ bovine insulin and 20% FBS [46]. MDA‐MB‐231‐luc2 [luciferase‐expressing MDA‐MB‐231 (RRID:CVCL_0062) cell line] cells strain D3H1 were obtained from Caliper Life Science (Hopkinton, MA, USA) and grown in DMEM medium with 10% FBS. All cell lines have been authenticated using deep sequencing analysis in the past 3 years. All experiments were performed with mycoplasma‐free cells.

### 2.7 Cell invasion assay for the tumorigenic breast cancer cell line MDA‐MB‐231‐luc2

The Boyden chamber invasion assay was performed as described previously [44], with the following modifications. The tumorigenic breast cancer cell line MDA‐MB‐231‐luc2 was cultured in a complete culture medium (i.e., DMEM medium with 10% FBS), grown to 70% confluence, harvested, and suspended in a serum‐free medium. Two hours before conducting the assay, inserts (ThinCert^Ò^ membranes with 8‐μm pores, Greiner BioOne, Kremsmünster, Austria) were coated with 30 μg of Matrigel (Corning, Corning, NY, USA). Cells (2.5 × 10^4^) were seeded on each insert placed in the upper chamber. A solution containing either APPI‐4M (10, 1, or 0.1 nm) or APPI‐3M (10, 1, or 0.1 nm), or PBS as a control, was added to cover each insert (to a total volume of 200 μL). The experiments for each condition were performed in triplicate. Culture medium (600 μL) with 10% FBS was used as a chemoattractant in the lower chamber. The Boyden chamber was then incubated in a CO_2_ incubator at 37 °C for 18 h. Following cell invasion, fluid and cells were removed from the upper chamber, and the inserts were fixed and stained with a Dipp Kwik Differential Stain kit (American Mastertech Scientific, Lodi, CA, USA). The invading cells, which had accumulated on the lower side of the insert, were counted (five fields per insert) with an EVOS FL cell imaging system (ThermoFisher Scientific, Waltham, MA, USA) at a magnification of ×10.

### 2.8 Cell viability assay for the tumorigenic breast cancer cell line MDA‐MB‐231‐luc2

Cell viability was evaluated by using an XTT cell proliferation kit (Biological Industries, Kibbutz Beit‐Haemek, Israel), according to the manufacturer's instructions. Briefly, MDA‐MB‐231‐luc2 cells were cultured in culture medium, grown to 70% confluence, harvested, and resuspended in a serum‐free medium; 2.5 × 10^4^ cells were then seeded in each well of a 96‐well plate. APPI‐4M, APPI‐3M (10, 1, or 0.1 nm), or PBS as a control, was added to each well to a total volume of 200 μL. The plate was incubated in a CO_2_ incubator at 37 °C for 18 h, and cell viability was determined according to the manufacturer's instructions. The experiment was conducted in triplicate.

### 2.9 Animals

Mice of different strains (see below) were obtained from Envigo (Jerusalem, Israel) and maintained according to the guidelines of the Ben‐Gurion University of the Negev (BGU) Animal Care Committee. Mice were housed in a dedicated pathogen‐free facility with a 12 h light/dark cycle. Food and water were provided *ad libitum*. Food, water, and bedding were sterilized, and cages were changed at least once a week.

### 2.10 *In‐vivo* clearance studies

BALB/c female mice (*BALB/cAnNCrl*, 6 weeks old) were injected intravenously (i.v.) with 200 μL of 1.5 mg·mL^−1^ APPI proteins, and serial blood samples were drawn from the tail vein over 6 h (at 1 h intervals) for APPI‐3M (or APPI‐4M), and over 94 h (at 1 and 24 h intervals) for APPI‐3M‐HSA (or APPI‐4M‐HSA), for 4 mice at each time point. Serum was separated using Minicollect® (Greiner, Germany) according to the manufacturer's protocol. To evaluate the serum bioavailability after subcutaneous (s.c.) administration of APPI‐3M‐HSA and APPI‐4M‐HSA, the proteins were also injected into mice subcutaneously (s.c.), 200 μL of 1.5 mg·mL^−1^ APPI protein solution, and serial blood samples were drawn from the tail vein over 94 h, for 4 mice at each time point.

Protein concentration in the serum was measured using a sandwich ELISA assay in a high‐binding 96‐well plate (Greiner, Germany). The plate was coated with 2 μg·mL^−1^ of purified KLK6 in coating buffer (100 mm bicarbonate, 35 mm carbonate, pH 9.5) overnight at 4 °C. The KLK6 solution was discarded, wells were washed three times with PBST (PBS with 0.05% Tween‐20) and blocked with 5% FBS for 1 h at room temperature. The FBS solution was discarded, wells were washed three times with PBST, and the serum samples containing APPI proteins were added in duplicate for each sample and incubated overnight at 4 °C. A standard curve was generated by making serial dilutions of APPI proteins [10–700 ng for APPI‐3M and APPI‐4M and 50–1000 ng for APP‐3M‐HSA and APPI‐4M‐HSA, in mouse serum (Sigma‐Aldrich)]. Wells were washed three times, and a detection antibody, 0.25 μg·mL^−1^ HRP‐conjugated anti‐6 × His tag (Abcam, Cambridge, UK), was added. After 2 h of incubation at room temperature, wells were washed three times, an HRP substrate, ultra‐TMB (3,3′,5,5′‐tetramethylbenzidine), was added, and incubation was continued until color appearance. A stop solution of 0.5 m H_2_SO_4_ was added, and the chromogenic reactions were determined using a plate reader (Biotek, Winooski, VT, USA) at 450 and 650 nm. The signals obtained for the 650 nm reading were subtracted from the 450‐nm signals. A graph of serum concentration vs. time was plotted, and the non‐compartmental pharmacokinetic analysis of the data was performed using the pksolver 2.0 Microsoft Excel add‐on [47].

### 2.11 Orthotopic model of prostate cancer spontaneous metastasis

Six‐ to 10‐week‐old Hsd:Athymic Nude‐Foxn1NU male mice were used for the orthotopic implantation experiment. PC3‐M Red‐FLuc cells were grown to 80% confluence, and cells were trypsinized and resuspended in 40% growth factor‐reduced Matrigel and 60% serum‐free RPMI‐1640 medium. Mice were anesthetized with an intraperitoneal (i.p.) injection of a solution of 100 mg·kg^−1^ ketamine and 10 mg·kg^−1^ xylazine. A small vertical incision was made in the lower abdominal region, and the prostate was exteriorized by exposing the bladder and seminal vesicle. PC3‐M cells (3 × 10^4^ cells in 10 μL) were injected into the dorsolateral side of the prostate using a 27‐gauge needle attached to a 100 μL glass syringe (100F‐LL‐GT, SGE). The peritoneum and skin were closed with sutures, and the mice were allowed to recover. Mice were randomized based on the baseline bio‐luminescence signal prior to assigning them to treatment cohorts. One week after PC3‐M cell transplantation, mice were injected daily with APPI‐3M, either 20 mg·kg^−1^ i.p. or 5 mg·kg^−1^ s.c., or with PBS as a control; 6 mice per group. At the treatment endpoint, i.e., on day 42, all mice were weighed and injected i.p. with 150 mg·kg^−1^ luciferin 10–15 min before they were euthanized. Mice organs were harvested, weighed, and scanned for bioluminescence using IVIS Lumina (PerkinElmer) to determine the signal intensity of the metastases and primary tumor (exposure time 2 s, binning 2, f‐stop 1.2). To detect fluorescently labeled APPI‐3M within the mouse organs, 200 μL of 2 nm of APPI‐3M, labeled with Alexa Fluor® 680 (according to manufacturer's protocol, 1 : 10 APPI : dye molar ratio), were injected i.v. 2 h before imaging. All bio‐imaging photographs presented in the Results section were captured under the same conditions (including the exposure times).

### 2.12 Orthotopic model of breast cancer spontaneous metastasis

MDA‐MB‐231‐LUC cells were cultured to 70% confluence, and 2 × 10^6^ cells in 100 μL PBS, were implanted s.c. into the mammary fat pad of 19 female 6‐week‐old NSG (NOD.Cg‐Prkdc^scid^Il2rg^tm1Wjl^/SzJ) female mice. Mice were then injected s.c. twice a week (starting from the day of cell injection) with 82.5 mg·kg^−1^ APPI‐3M‐HSA (10 mice per group) or with PBS (9 mice per group) as a control. Once a week, mice were injected i.p. with 150 mg·kg^−1^ luciferin, and tumor and metastasis formation were followed using NEWTON 7.0 (Vilber, Collégien, France). At the treatment endpoint, on day 25, all mice were injected i.p. with 150 mg·kg^−1^ luciferin 10–15 min before they were euthanized. Mice organs were then harvested and scanned for bioluminescence using NEWTON 7.0 to evaluate the metastatic burden (exposure time 30 s, binning 2, f‐stop 0.7). In addition to metastatic potential, the *in‐vivo* local effect of treatment was assessed qualitatively both macroscopically and microscopically, i.e., in terms of the ease of surgical excision of the primary tumor bulk and histopathological evidence (see next section) of muscle invasion, respectively.

### 2.13 Immunohistochemistry staining

Lung tissues were fixed in 4% paraformaldehyde solution overnight at room temperature and then maintained in 70% ethanol until embedding in paraffin. Paraffin‐embedded tissue blocks were sectioned into 5‐μm slices, loaded onto microscope slides, and incubated at 60 °C for 1 h. After deparaffinization with a xylene substitute (3803672E, Leica, Deer Park, IL, USA) and rehydration steps in a descending alcohol series, antigen retrieval was performed. The slides were incubated in 10 mm citric acid buffer, pH 6.0, at 100 °C for 15 min, then cooled in buffer at room temperature and washed three times with distilled water for 3 min. Endogenous peroxidases were inactivated with 0.3% hydrogen peroxide in methanol buffer for 30 min. The slides were washed three times with PBS 1% for 3 min and then blocked with 5% BSA in PBS‐T (0.1% Tween) for 1 h at room temperature. Thereafter, the slides were incubated overnight at 4 °C with the primary antibody, anti‐KRT14 (1 : 1000, Abcam, EPR17350). The following day, the slides were washed three times with PBS‐T. The ABC kit (VECTASTAIN® ABC, Newark, CA, USA) was used for detection according to the manufacturer's protocol, with 3,3′‐diaminobenzidine (DAB, ScyTek Laboratories, Logan, UT, USA) as a substrate for color development. Slides were counterstained with hematoxylin, dehydrated, and mounted in mounting medium (Sub‐X, Leica 3801740). All slides were digitalized using the Panoramic Scanner (3DHISTECH, Budapest, Hungary), and the analysis was performed with caseviewer software (3DHISTECH). In this experiment, four lungs were analyzed with 8–10 random areas per mouse, and a *T*‐test was used for statistical analysis.

### 2.14 Orthotopic model of ovarian cancer spontaneous metastases

OVCAR‐3‐LUC cells were cultured to 70% confluence. Tumor implantation was performed on six‐week‐old severe combined immunodeficient (SCID) female mice, namely NOD.Cg‐*Prkdc* ^*scid*^ *Il2rg* ^*tm1Wjl*^/SzJ (NSG, NOD *scid* gamma), as described previously [48]. A two‐step process was used for orthotopic tumor implantation; first, tumors were generated by injecting s.c. 2 × 10^6^ OVCAR‐3‐LUC cells in 50 μL of Hanks balanced salt solution to the flanks of SCID female mice. After 120 days, when the tumors had reached a size of 100–200 mm^3^, they were excised and cut into 1 mm^3^ pieces. Second, orthotopic implantations were performed in different groups of SCID female mice by stitching a single tumor piece onto the right ovary of each mouse. One day after the orthotopic implantations, the mice were injected s.c. twice a week with 50 mg·kg^−1^ APPI‐4M‐HSA (7 mice per group) or PBS (8 mice per group). Once a week, mice were also injected i.p. with 150 mg·kg^−1^ luciferin, and tumor and metastasis formation were followed using NEWTON 7.0 (Vilber). At the treatment endpoint, on day 30, all mice were injected i.p. with 150 mg·kg^−1^ luciferin 10–15 min before they were euthanized. Mice organs were then harvested and scanned for bioluminescence using NEWTON 7.0 to determine the signal intensity of the metastases and primary tumors. To detect fluorescently labeled APPI‐4M in mouse organs, 200 μL of 2 nm APPI‐4M, labeled with Alexa Fluor® 680 (labeled according to manufacturer's protocol, 1 : 10 APPI : dye molar ratio), were injected i.v. 2 h before imaging (exposure time 1 s).

### 2.15 Statistical analysis

The results are presented as the means ± SD or ±SEM. Statistically significant differences between the groups were revealed using one‐way ANOVA analysis, followed by Tukey's multiple comparison test.

## 3 Results

### 3.1 Fluorescently labeled APPI‐3M accumulates in prostate cancer tumors and metastatic sites

**Fig. 1:**
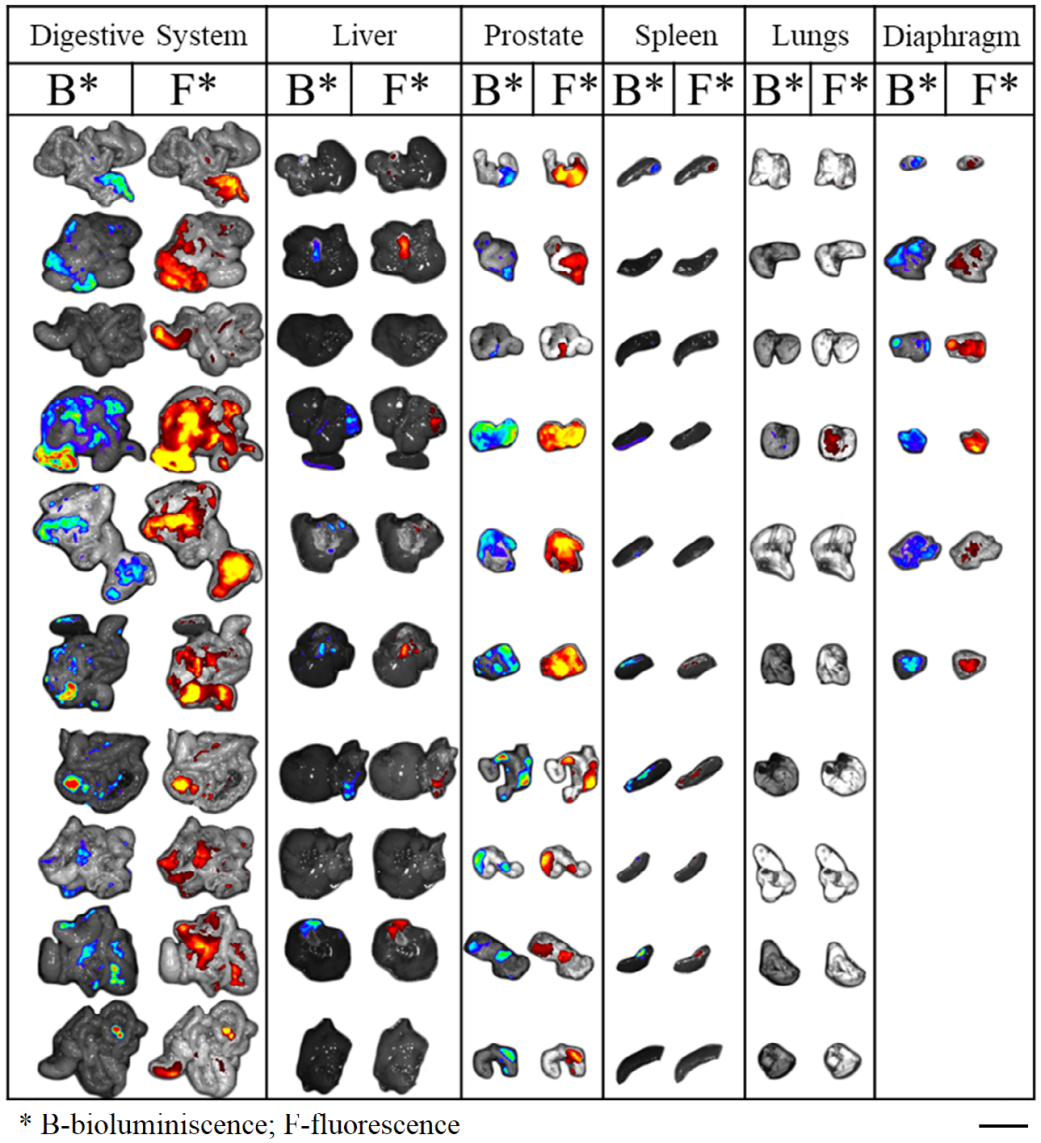
Fluorescently labeled APPI‐3M (APPI‐3M_K29L_‐AF‐680) binds to PC3‐M prostate primary tumor and metastatic sites *in vivo*. PC3‐M tumor‐bearing mice were injected i.v. with 2 nmol of APPI‐3M_K29L_‐AF‐680, and 4 h later an i.p. injection of 150 mg·kg^−1^ luciferin was administered. Mouse organs were harvested and scanned for luminescence and fluorescence using IVIS to evaluate protein accumulation in metastases and primary tumors. Colored signals under F correspond to the fluorescence signal originating from APPI‐3M_K29L_‐AF‐680. Colored signals under B indicate bioluminescence of PC3‐M cells. *n* = 10. The scale bar is 1 cm.

We have previously demonstrated [25] that APPI‐3M acts as a potent and selective inhibitor in cell‐based models of mesotrypsin‐dependent prostate cancer cell invasion and spread. To further explore the therapeutic potential of APPI‐3M, we used an orthotopic mouse model of the highly metastatic PC3‐M human prostate cancer, whose invasiveness has previously been shown to be mesotrypsin dependent [9]. To this end, luciferase‐transfected PC3‐M cells were injected into the prostate of athymic nude mice, leading to the generation of metastases within 3–6 weeks, as detected by whole body (or dissected organs) measurements of luciferin bioluminescence. To investigate whether APPI‐3M accumulates in primary tumors within the prostate, we performed an identical experiment with an APPI‐3M variant that had been site‐specifically labeled at K29L by coupling the Alexa Fluor‐680 fluorescent dye to the single primary amine at the N‐terminus (via an NHS‐ester reaction). The *K*_*i*_ and *K*_cat_ values for the Alexa Fluor‐680‐labeled protein, designated APPI‐3M_K29L_‐AF‐680, were (8.9 ± 0.1) × 10^−11^ m and (5.3 ± 1.0) × 10^−4^ s^−1^, respectively—values that are similar to those obtained for unlabeled APPI‐3M [25]. Whole‐body optical imaging analysis showed that 10 min after i.v. injection, APPI‐3M_K29L_‐AF‐680 had spread homogeneously within the mouse body, reaching all organs. Notably, by allowing 4 h for protein clearance from the body, strong binding of APPI‐3M_K29L_‐AF‐680 to PC3‐M cells in both primary tumor and distant metastases was observed (Fig. 1), with residual unbound protein accumulating in the kidneys.

### 3.2 APPI‐3M inhibits prostate cancer metastases in an orthotopic model of spontaneous metastasis *in vivo*

**Fig. 2:**
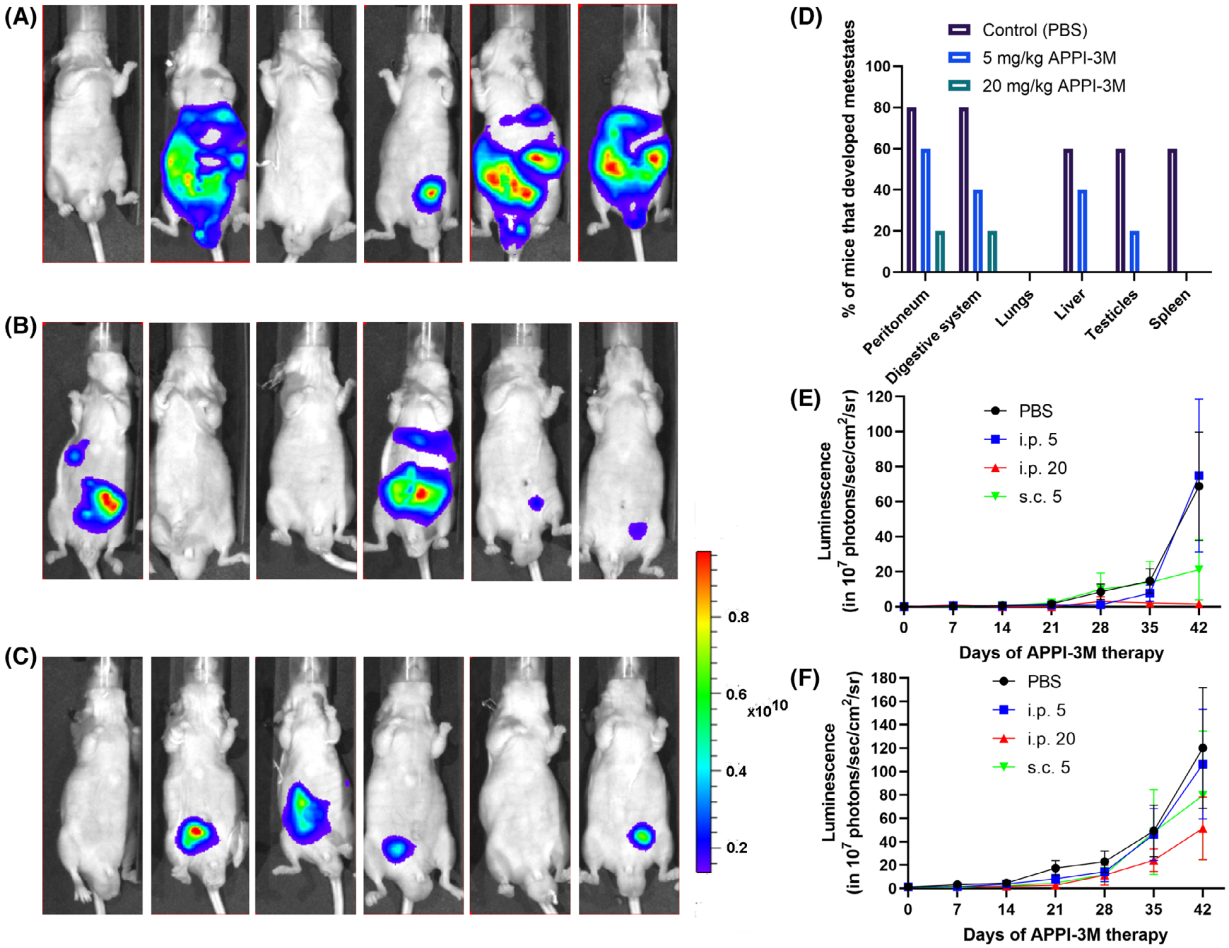
APPI‐3M treatment reduced primary tumor growth and metastasis formation in an orthotopic prostate cancer model. Starting 1 week after cancer cell injection, mice were treated daily for 7 weeks with APPI‐3M – either s.c. injections of 5 mg·kg^−1^ or i.p. injections of 5 or 20 mg·kg^−1^ – or with PBS as control. Whole‐body luciferase bioluminescence was measured on day 42 by using IVIS. (A–C) Results are shown for (A) the control group, injected only with PBS; (B) the group injected with APPI‐3M, 5 mg·kg^−1^ s.c.; and (C) the group injected with APPI‐3M, 20 mg·kg^−1^ i.p. (*n* = 5) (D) On day 42, selected organs were harvested and scanned for bioluminescence by using IVIS. Based on IVIS scans and visual examination, the presence of metastases was determined for each mouse (*n* = 5). A single mouse with no luminescence signal (whole‐body imaging, panel C) developed a primary tumor (observed upon organ removal). (E, F) Whole‐body luminescence scans of live mice were performed weekly to track changes in the numbers of metastases (E) and primary tumors (F). Each point on the graphs represents the mean luminescence measurement per day ± SEM (*n* = 6). *Y*‐axis scale represents the luminescence signal intensity to the power of 10^7^.

To test the therapeutic potential of APPI‐3M *in vivo*, the above‐described orthotopic mouse model of human PC3‐M prostate cancer cells was used. One week after PC3‐M cell transplantation, mice were injected daily with the following doses of APPI‐3M: 5 or 20 mg·kg^−1^, i.p. or 5 mg·kg^−1^, s.c.; a control group was injected only with PBS (Fig. 2). Notably, APPI‐3M treatments at a dose of 20 mg·kg^−1^ i.p. or 5 mg·kg^−1^ s.c. showed the most potent effects in inhibiting metastases and primary tumors, as demonstrated by monitoring the whole‐body bioluminescence signal of PC3‐M cells over time (Fig. 2E,F); in particular, at the treatment endpoint (day 42), the 20 mg·kg^−1^ i.p. treatment had led to a 98% and 57% reduction in the numbers of metastases and primary tumors, respectively. At the treatment endpoint, all mice were injected i.p. with 150 mg·kg^−1^ luciferin (10–15 min before euthanasia), and their organs were harvested, weighed, and scanned for bioluminescence using IVIS to evaluate metastases and primary tumor signal intensities *ex vivo* (Fig. S1). The *ex vivo* results for PC3‐M bioluminescence were consistent with whole‐body imaging, particularly with the results obtained from the i.p. and s.c. treatments (Fig. 2) at doses of 20 and 5 mg·kg^−1^, respectively; these results showed a lower, but not statistically significant, bioluminescence signal in all organs tested, compared to the control (PBS), and demonstrated an inhibitory effect on the formation of both metastases and primary tumors. No major differences in total or organ weights were recorded upon treatment with APPI‐3M (Fig. S2).

### 3.3 APPI‐3M inhibits invasiveness of metastatic breast cancer cells

**Fig. 3:**
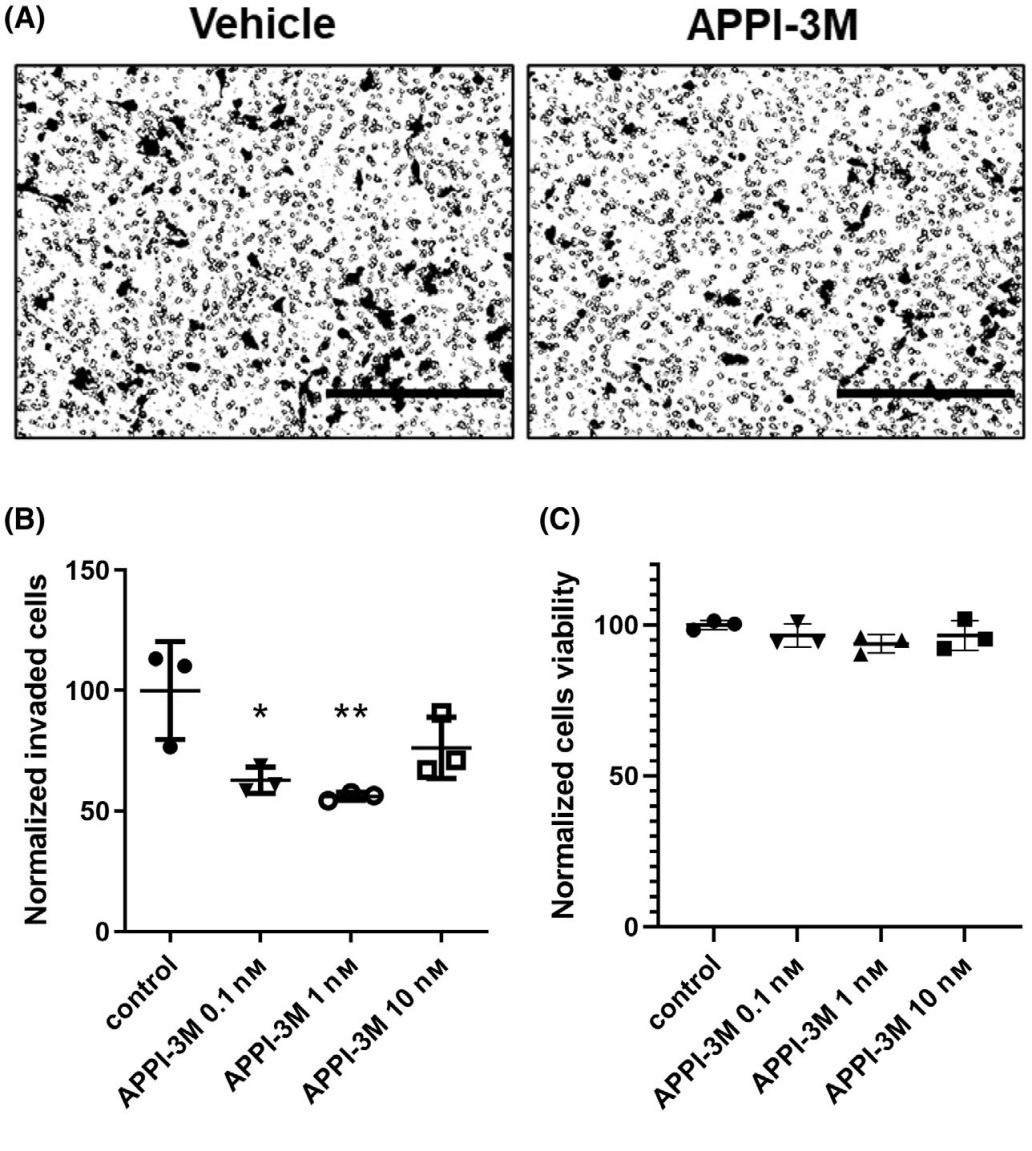
APPI‐3M reduces the invasiveness but not the viability of MDA‐MB‐231 breast cancer cells. The invasive ability of MDA‐MB‐231 cells in the presence of APPI‐3M was evaluated using a Matrigel‐coated Boyden chamber. The cells that successfully invaded the Matrigel 18 h after plating were stained and quantified. (A) Representative images of the invading cells treated with either vehicle or APPI‐3M (100 pm). Scale bars, 400 μm. (B) Quantification of invading cells, normalized to control. (C) Quantification of cell viability using the XTT assay, 18 h after incubation with the vehicle or APPI‐3M (at 0.1 nm, 1 nm and 10 nm), normalized to control. For panels (B and C), *T*‐test was used for statistical analysis. The data are presented as means ± SD of three biological replicates. *P < 0.05; **P < 0.01 compared to the control group.

Previously, we demonstrated the ability of APPI‐3M to inhibit the migration of prostate cancer cells [25]. In the present study, we complemented the data by testing the ability of APPI‐3M to inhibit the invasive behavior of mesotrypsin‐expressing metastatic human MDA‐MB‐231 breast cancer cells [46]. To this end, we used a Boyden chamber assay, in which the cells invade through a Matrigel‐coated membrane, and their accumulation on the membrane is visualized by microscopy. We found that upon treatment with 0.1, 1, or 10 nm APPI‐3M, MDA‐MB‐231 cells showed a significant decrease (albeit with an inverted dose‐dependent trend) in invasiveness (up to 44%) through the Matrigel, as compared to the untreated control (Fig. 3A,B). Notably, in an XTT viability assay (Fig. 3C), APPI‐3M did not affect the viability of MDA‐MB‐231 cells. These results indicate that the observed decrease in the number of invading cells in the Boyden chamber assay was not due to cell death but rather due to the ability of APPI‐3M to inhibit cell invasiveness.

### 3.4 PEGylation of APPI results in low production yield

To improve the pharmacokinetic profile of APPI‐3M or APPI‐4M (a potent KLK6‐targeting inhibitor [44]), we increased the size of both proteins by PEGylating them with methoxy PEG succinimidyl carboxymethyl ester (MW 20 kDa). Since both APPI proteins have four reactive amines (one at the N‐terminus, 2 lysines at the FLAG tag, and another lysine at position 29), we expected that the PEGylation reaction would result in a mixture of five different products, with each APPI variant having zero to four conjugated PEG units. However, an analysis of the reaction products (using SDS/PAGE) showed a loss of > 50% of APPI‐3M or APPI‐4M in the PEGylation process (during mounting on nickel beads and the multiple washing steps required for this procedure). For the APPI‐3M material that was recovered, the total PEGylation yield was 72% – with 55% labeled in one position, 12% in two positions, 2.8% in three positions, and 2.3% in four positions (Fig. S3A) – leaving a significant fraction (28%) of APPI‐3M unreacted. In contrast, PEGylation of APPI‐4M resulted in 100% labeling (of the recovered material). *K*_*i*_ measurements for APPI‐3M and APPI‐3M‐PEG showed similar *K*_*i*_ values of 469.0 ± 73.4 and 376.9 ± 24.0 pm, respectively (Fig. S4).

### 3.5 Fusion to human serum albumin improves the pharmacokinetic profile of APPI

**Fig. 4:**
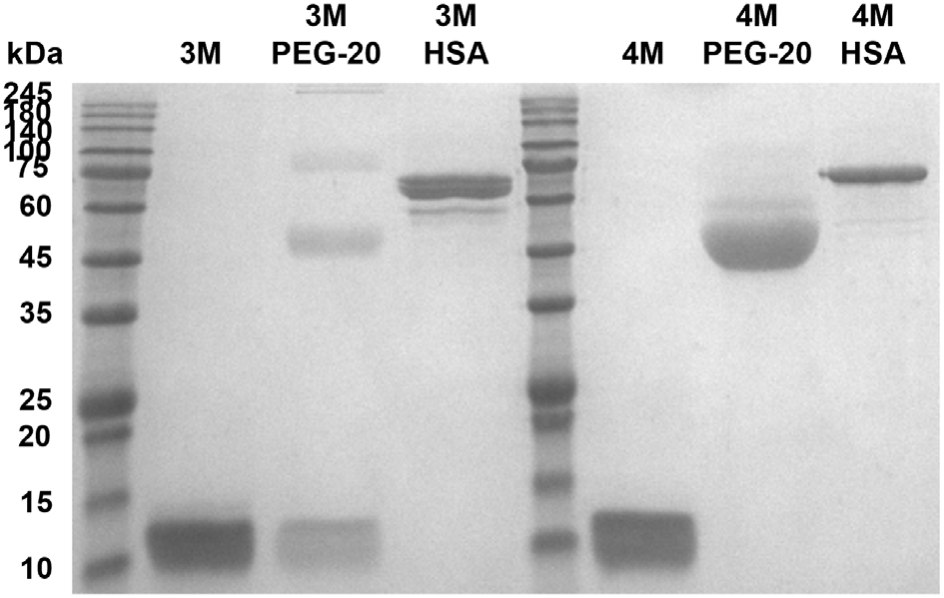
SDS/PAGE analysis of PEGylated APPI‐3M and APPI‐4M products pre‐ and post‐ size exclusion chromatography (SEC), respectively. APPI‐3M and APPI‐4M were either PEGylated with 20 kDa PEG or fused to human serum albumin (HSA). Purity of the APPI‐3M and APPI‐4M products was above 95%, as determined from PAGE analysis (*n* = 3).

Due to the low yield of the PEGylated APPI proteins, we decided to increase the molecular weight of APPI by fusion with HSA. HSA fusion to APPI‐3M or APPI‐4M yielded proteins with a molecular weight of 77 kDa (Fig. 4 and Fig. S3). Yields of purified APPI‐3M‐HSA and APPI‐4M‐HSA produced in a bioreactor were high (40–80 mg·L^−1^ medium). Ki measurements for APPI‐3M and APPI‐3M‐HSA showed similar *K*_*i*_ values of 469.0 ± 73.4 and 626.0 ± 304.2 pm, respectively, meaning that fusion to HSA does not disrupt inhibition potency of APPI‐3M. To determine whether HSA fusion improved the pharmacokinetic profile of APPI, we injected APPI‐3M, APPI‐4M, and their HSA fusions (APPI‐3M‐HSA and APPI‐4M‐HSA, respectively) i.v. into naïve mice and followed the serum levels over time.

**Fig. 5:**
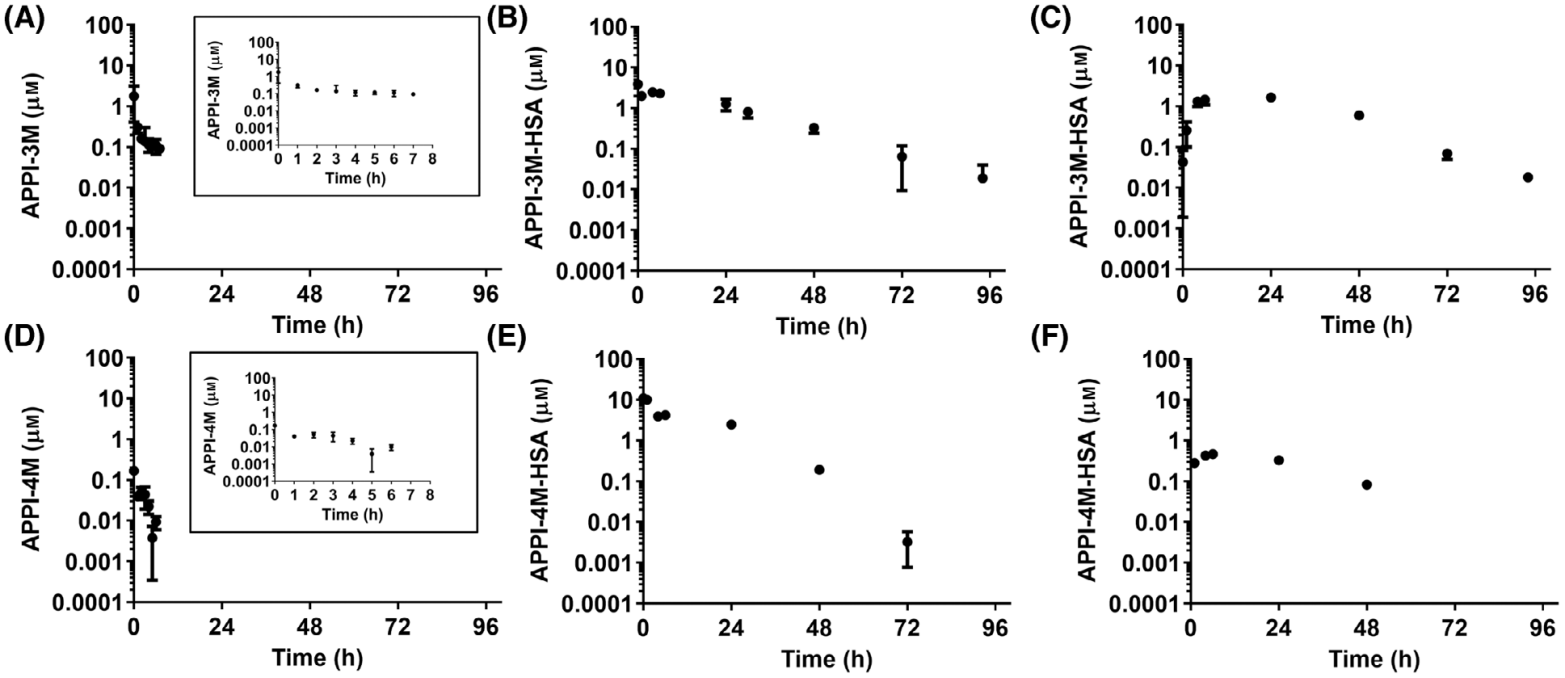
*In‐vivo* serum concentration vs. time curves of APPI‐3M, APPI‐3M‐HSA, APPI‐4M and APPI‐4M‐HSA in naïve mice. APPI was characterized by rapid elimination from the central circulation and low AUC values, while APPI‐HSA fusion proteins were detected in the serum over more than 90 h. Mice (6 weeks old) were injected i.v. (A, B, D, E) or s.c. (C, F) with 300 μg APPI‐3M, APPI‐3M‐HSA, APPI‐4M or APPI‐4M‐HSA, and blood samples were collected at different time points. APPI serum concentrations were determined by ELISA, using KLK6 as the capturing agent. The data are means ± SD (*n* = 4). Inset, *in vivo* serum concentrations vs. time during the first 8 h.

HSA fusion induced a substantial increase in the systemic exposure of APPI‐3M‐HSA and APPI‐4M‐HSA, as compared to the original proteins (Fig. 5 and Table S1). Following i.v. administration, the concentrations of the fusion proteins were higher than those of the original proteins over the entire time course of blood sampling. Of note, the fusion proteins were given at an ~ 7 times lower molar concentration than the original proteins (i.e., all proteins were given at 300 μg per injection, but the molecular weight of the fusion proteins is ~ 7 times higher than that of the original proteins). The APPI‐3M and APPI‐4M concentrations reached a plateau after 2 and 5 h, respectively, and then declined below the limit of quantitation after 7 and 6 h, respectively (Fig. 5A,D). In contrast, measurable, and substantial, concentrations of APPI‐3M‐HSA and APPI‐4M‐HSA were detected over 96 and 72 h after administration, respectively (Fig. 5B,E). Overall, based on the i.v. datasets, HSA fusion resulted in a 16.7‐fold and a 536‐fold increase in the area under the curve (AUC) values of APPI‐3M and APPI‐4M, respectively. However, the half‐life values were affected to a lesser extent by the HSA fusion (Table S1). Notably, APPI‐3M‐HSA was characterized by complete bioavailability after s.c. administration, as reflected in the AUC values (Table S1). In contrast, the bioavailability of APPI‐4M‐HSA after s.c. administration was substantially lower, by approximately 10.5% (Table S1). Nevertheless, s.c. administration of the HSA‐fused proteins resulted in appreciably higher concentrations and more extensive and prolonged exposure to these proteins than i.v. administration of the corresponding non‐fused proteins (Fig. 5 and Table S1). Therefore, in the subsequent *in‐vivo* experiments, APPI‐3M‐HSA and APPI‐4M‐HSA were administered s.c.

### 3.6 APPI‐3M‐HSA inhibits breast cancer metastasis in an orthotopic mouse model of spontaneous metastasis

**Fig. 6:**
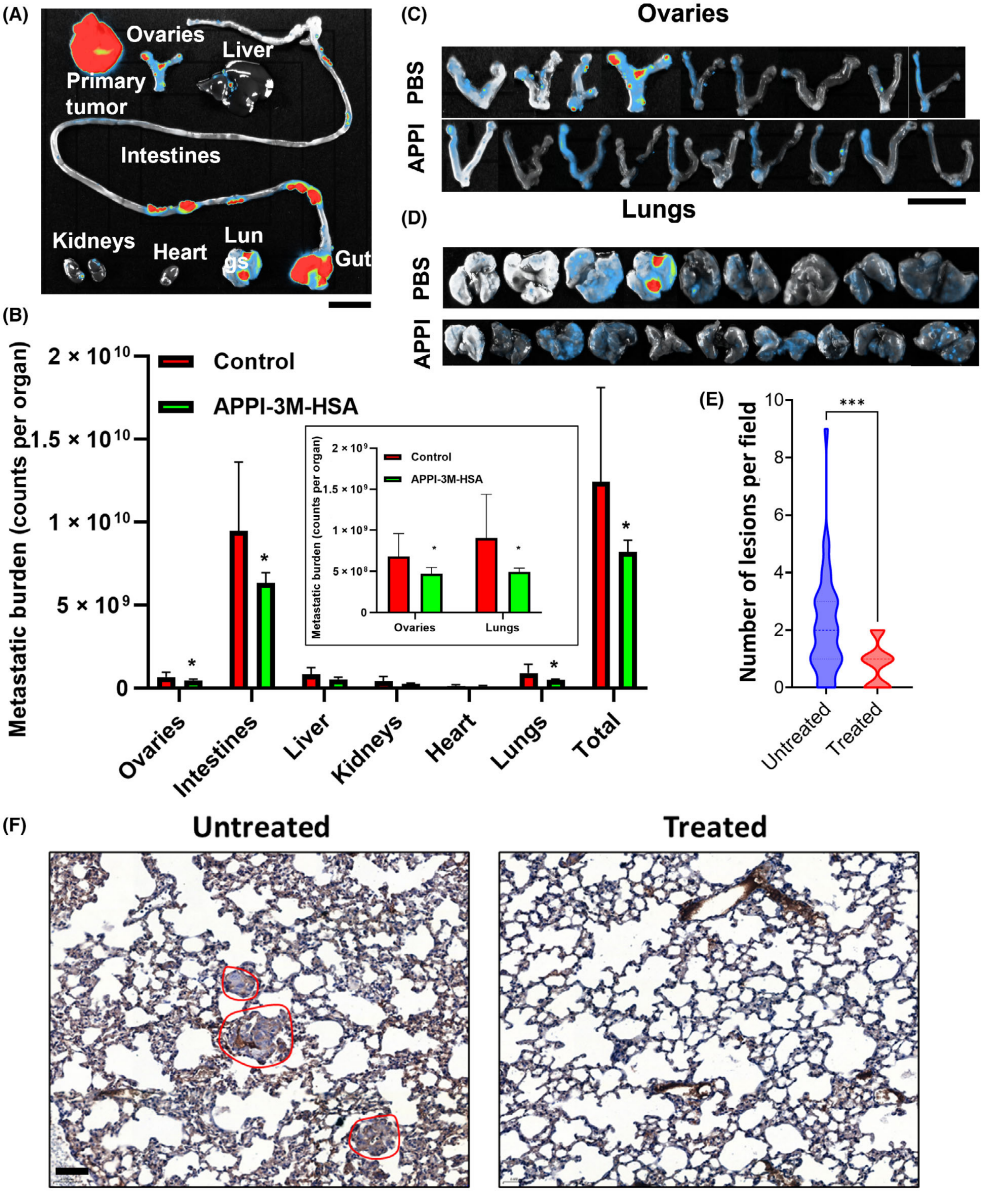
APPI‐3M‐HSA treatment reduced metastasis formation in an orthotopic breast cancer mouse model of spontaneous metastasis. Mice were treated for 25 days with twice weekly s.c. injections of APPI‐3M‐HSA (82.5 mg·kg^−1^) or PBS. (A) Selected organs were harvested and scanned for bioluminescence with the NEWTON 7.0 imaging system (*n* = 10). (B) Metastatic burden was measured for the control and APPI‐3M‐HSA‐treated groups. Images were taken separately for each organ, using a similar setup for all the images. Results are presented as means ± SD; *n* = 10 in the APPI‐3M‐HSA‐treated group, *n* = 9 in the control group. *P < 0.05. Inset – results for ovaries and lungs at higher magnification. (C, D) Ovaries and lungs of control and APPI‐3M‐HSA‐treated mice (measured on day 25). (E) Number of micrometastases in the lungs of tumor‐bearing mice treated with PBS (designated untreated) or APPI‐3M‐HSA. For panels (B and E), *T*‐test was used for statistical analysis (*n* = 10). ***P < 0.001 compared to the control group. (F) Representative images of micrometastases (marled with red circles) in the lungs of breast tumor‐bearing mice treated as indicated above (*n* = 10). The scale bar for panel (A) is 1 cm, for panels (B and C) is 1.5 cm and for panel (F) is 50 μm.

Since our results showed the potency of APPI‐3M in inhibiting the invasion of MDA‐MB‐231 breast cancer cells *in vitro* (Fig. 3), we sought to demonstrate its ability to inhibit MDA‐MB‐231 metastasis *in vivo*. For this purpose, we established an *in‐vivo* orthotopic model of human breast cancer using MDA‐MB‐231‐LUC tumor xenografts in immunodeficient mice [49]. The MDA‐MB‐231‐LUC cells were injected orthotopically into the mammary fat pads of NOD‐SCID‐IL2R γ^null^ (NSG) female mice. The mice were then treated twice a week with 82.5 mg·kg^−1^ APPI‐3M‐HSA (10 mice per group) or PBS (9 mice per group). The choice of the s.c. administration route and dosing frequency were based on the pharmacokinetic properties of APPI‐3M‐HSA, which was characterized by prolonged systemic exposure over 3–4 days after s.c. injection (Fig. 5C). The mice were imaged weekly after luciferin injection to follow primary tumor progression and metastasis formation (Fig. S5). The presence of metastatic sites was recorded at dissection 25 days after cell injection. To this end, ovaries, intestines, liver, kidneys, heart, and lungs were harvested and imaged (by luminescence imaging), and the metastatic burden was evaluated for each organ (Fig. 6A,C,D). The results showed a significant decrease in the metastatic burden in various organs of the APPI‐3M‐HSA‐treated mice (30% in the ovaries, 33% in the intestines, and 45% in the lungs) and a 34% reduction in total metastatic burden, compared with the PBS‐treated mice (Fig. 6B). There was no significant difference in the metastatic burden between the treatments in the liver, heart, and kidneys.

To provide support for the luminescence results, we conducted a histopathology analysis of the lungs of the MDA‐MB‐231 breast tumor‐bearing mice treated with APPI‐3M‐HSA or PBS. A count of the micrometastases (KRT14 positive cells) in the lungs indicated that treatment of mice with APPI‐3M‐HSA significantly reduced the number of metastatic lesions vs. the number in the lungs of tumor‐bearing mice treated with PBS (Fig. 6). We note here that in the APPI‐3M‐HSA‐treated group, the primary tumors could be easily separated from the adjacent structures and could be completely excised, while tumors of the untreated group were difficult to excise, and the specimens contained abundant muscular tissue (*n* = 4).

Microscopically, specimens of lungs from both treated and untreated groups contained sheets of highly malignant epithelial cells. In the treated group, invasion of striated muscle by neoplastic cells was observed only rarely; in those histopathology specimens, single uninjured striated muscle fibers were observed in an abundant neoplastic arena. In contrast, in the untreated group, abundant striated muscle tissue indicated intramuscular invasion by the malignant cells, both longitudinally and transversally. In addition, it appeared that the malignant cells invaded the muscle by first forming isolated cancerous islands within the muscle fibers and subsequently gradually completely replacing the muscle tissue (Fig. S6).

### 3.7 APPI‐4M‐HSA inhibits metastases in an orthotopic model of ovarian cancer

We previously demonstrated [44] that APPI‐4M acts as a functional inhibitor of KLK6‐dependent breast cancer cell migration and invasion. To further explore the therapeutic potential of APPI‐4M‐HSA in a physiologically relevant environment, we used an orthotopic model of human ovarian cancer comprising OVCAR‐3‐LUC tumor xenografts (express KLK6) in immunodeficient mice [48], where OVCAR‐3 cells are human epithelial ovary adenocarcinoma cells originally isolated from a malignant effusion. In a typical orthotopic model of ovarian cancer, ovarian cancer cells are injected into the peritoneal cavity, inducing the peritoneal spread of the tumor, which may not accurately mimic the pathological situation in which a primary solid tumor develops in the ovary. We therefore chose to perform an orthotopic implant of an ovarian cancer tumor onto the right ovary of SCID female mice.

**Fig. 7:**
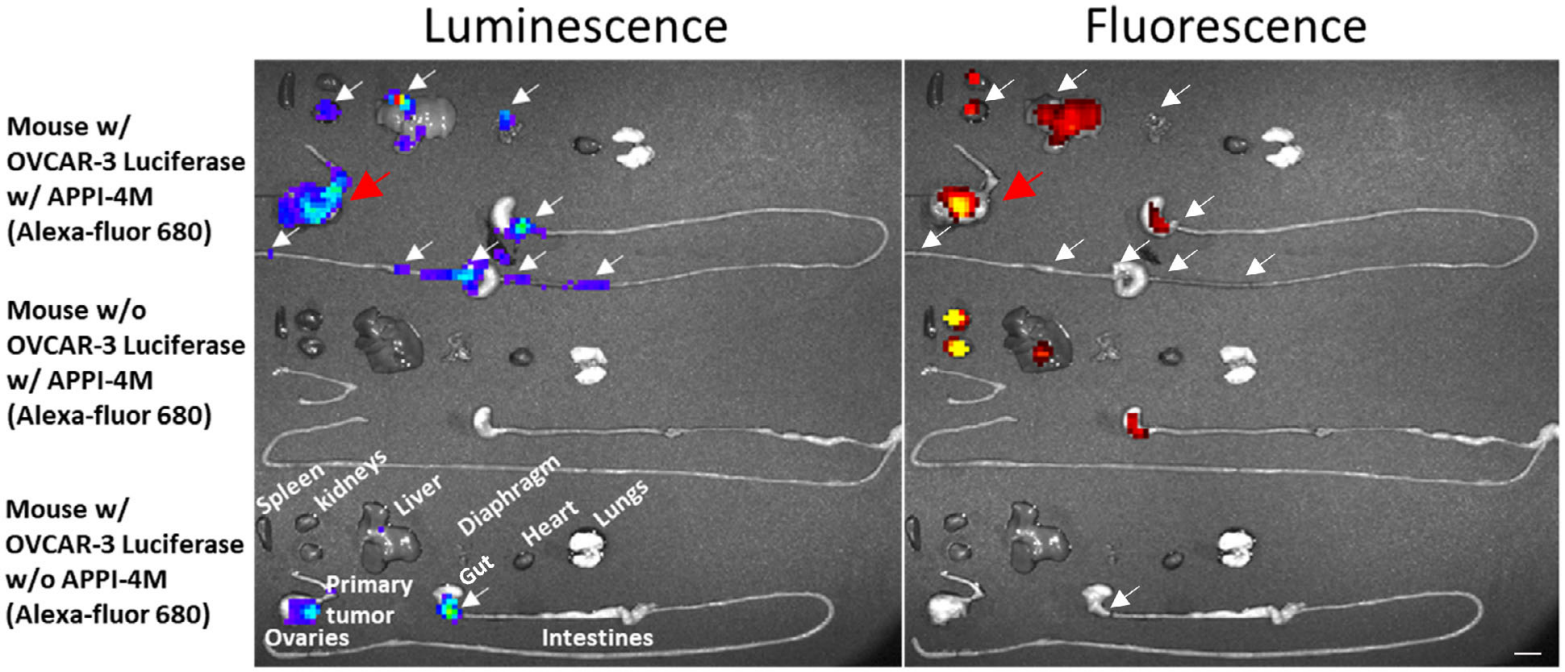
Fluorescently labeled APPI‐4M accumulated in the KLK6‐expressing OVCAR‐3 ovarian primary tumors (indicated with red arrows) but not in the metastatic sites (indicated with white arrows). OVCAR‐3‐LUC‐tumor‐bearing mice were injected i.v. with 1 nmol of APPI‐4M‐AF‐680, followed (2 h later) by i.p. injection of 150 mg·kg^−1^ luciferin. Mouse organs were then harvested and scanned for luminescence and fluorescence by using IVIS to probe the metastatic and primary tumor signal intensities. Controls were tumor‐free mice that had been injected with 1 nmol of APPI‐4M‐AF‐680 and OVCAR‐3‐LUC‐tumor‐bearing mice that had been injected with PBS (*n* = 6). The scale bar is 1 cm.

The model was first used to test whether APPI‐4M accumulates in ovarian tumors and/or reaches metastatic sites. To this end, we conjugated APPI‐4M to the Alexa Fluor‐680 fluorescent dye (via an NHS‐ester reaction) and injected the conjugate i.v. into OVCAR‐3‐LUC‐tumor‐bearing mice. After 2 h, to allow for protein clearance from the body, specific binding (measured from an overlay of the fluorescence and bioluminescence signals) of the protein to OVCAR‐3‐LUC cells was observed for the primary tumor (Fig. 7). The remainder of the protein (not bound to OVCAR‐3‐LUC cells, i.e., Alexa Fluor‐680 fluorescence positive and luminescence negative) accumulated in the kidneys, liver, and gut.

**Fig. 8:**
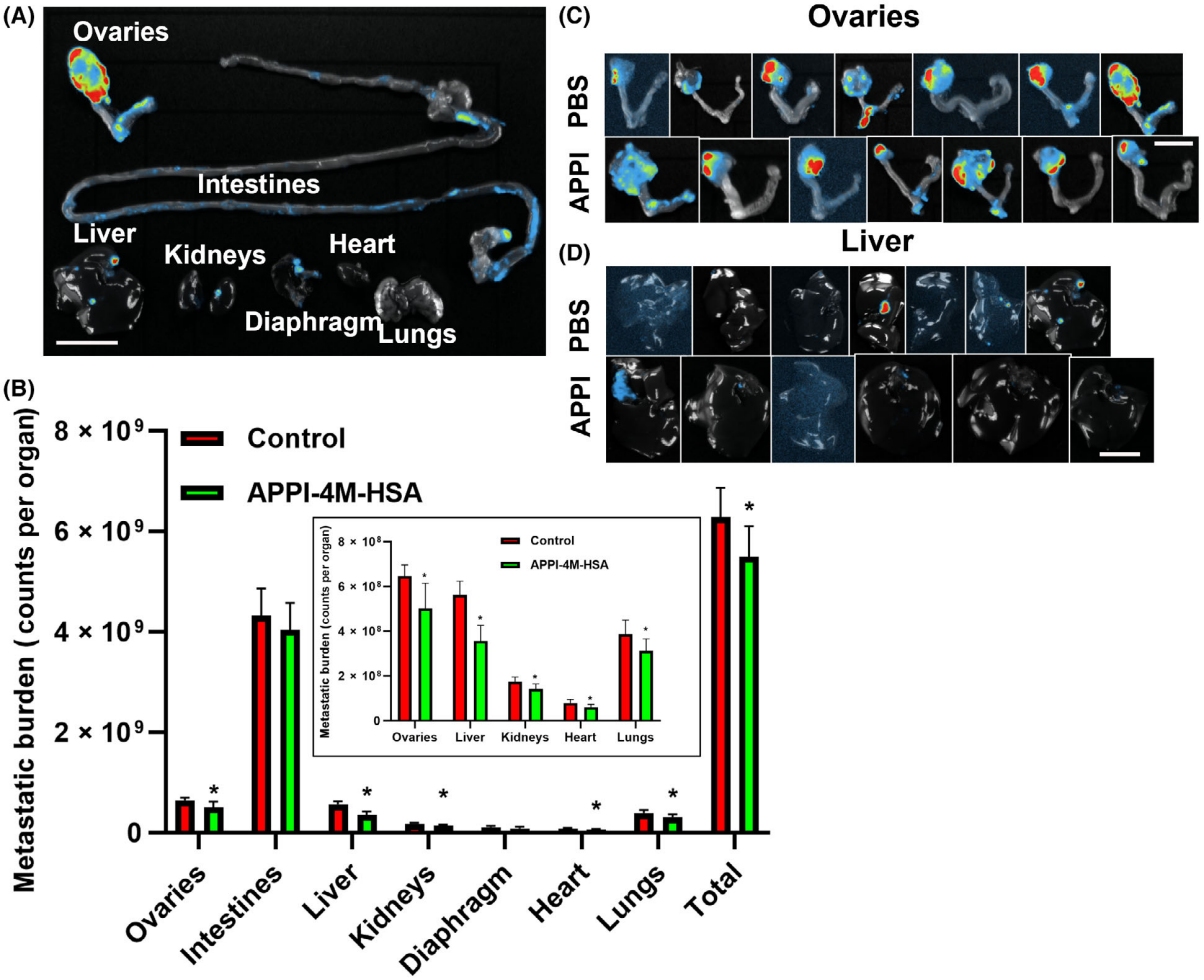
APPI‐4M‐HSA treatment reduced primary tumor progression and formation of metastases in an orthotopic ovarian cancer model. OVCAR‐3‐LUC tumor‐bearing mice were treated for 30 days with twice weekly s.c. injections of 50 mg·kg^−1^ APPI‐4M‐HSA or PBS. (A) Selected organs of untreated OVCAR‐3‐LUC tumor‐bearing mice were harvested and scanned for bioluminescence by using the NEWTON 7.0 imaging system (*n* = 6). (B) Metastatic burden was determined for each group of mice (control and APPI‐4M‐HSA treated). Images were taken separately for each organ using a similar setup for all the images. The data are means ± SD (*n* = 6). *T*‐test was used for statistical analysis. *P < 0.05. Inset – results for ovaries and lungs at an expanded scale. (C, D) Ovaries and livers of control and APPI‐4M‐HSA treated mice (*n* = 6). Red and blue represent high and low fluorescent signals, respectively. The scale bar for panel (A) is 1.5 cm and for panels (C and D) is 1 cm.

To test the therapeutic potential of APPI‐4M‐HSA *in vivo*, the same orthotopic mouse model of human ovarian OVCAR‐3‐LUC cancer was used. Seven mice per group were injected s.c. twice a week with 50 mg·kg^−1^ of APPI‐4M‐HSA or with PBS (Fig. 8A–D). This dose was somewhat lower than that for APPI‐3M‐HSA (see the previous section) and was expected to result in lower systemic exposure to the HSA‐fused protein (see the pharmacokinetic data in Fig. 5 and Table S1). Nevertheless, we judged that this dose would be sufficient for the anti‐metastatic activity of APPI‐4M‐HSA, which was characterized by prolonged systemic exposure over at least 2 days after s.c. administration (Fig. 5F). After luciferin injection, mice were imaged weekly to follow primary tumor progression and metastatic spread in the peritoneum. Primary tumor and metastatic sites were recorded at dissection 30 days after cell implantation. Ovaries, intestines, liver, kidneys, diaphragm, and lungs were harvested and imaged, and the tumor and metastatic burden were evaluated for each organ (Fig. 8A,C,D). The results showed a significant decrease in the primary tumor and metastatic burden in the treated mice compared with control untreated mice, namely, a drop of 22% in the tumor/metastatic burden in the ovaries, 36% in the liver, 19% in the kidneys, 23% in the heart, of 19% in the lungs and 13% in total burden. There was no significant decrease in the tumor/metastatic burden in the intestines or the diaphragm (Fig. 8B).

## 4 Discussion

Encouraged by our early *in‐vitro* studies [25, 44] demonstrating the high potency of APPI‐3M in targeting mesotrypsin and of APPI‐4M in targeting KLK6, in the present work we further developed these inhibitors toward clinical use. To this end, we fused each inhibitor to HSA to improve its pharmacokinetic properties, such as bioavailability and clearance after s.c. injection. Since all APPI proteins are resistant to hydrolysis by KLK6, we could use it as a “capturing agent” in an ELISA assay to follow protein plasma levels and pharmacokinetic profile. In addition, since KLK6 can bind only active (uncleaved and correctly folded) APPI, our ELISA experimental setup can be used to quantify APPI levels in mouse serum and to accurately detect the quantity of functional intact APPI. This new experimental setup for pharmacokinetic analysis may lead to the development of other *ex‐vivo* assays for measuring serum concentrations of other endogenous targets (e.g., protease inhibitors) in their active form. The pharmacokinetic results obtained using this experimental setup confirmed that the structural stability and resistance to cleavage by proteases were not impaired in circulating APPI proteins fused to HSA. In addition, our results showed that s.c. administered APPI‐3M‐HSA exhibited 100% bioavailability and an extended half‐life in the circulation relative to APPI‐3M lacking HSA. These properties (bioavailability and extended half‐life) are superior to those of several other peptide‐based therapeutics [50], which some of them tend to undergo substantial metabolism at the s.c. injection site and rapid clearance, e.g., via proteolysis or renal elimination. Thus, our new HSA‐fused proteins possess inherent stability and proteolytic resistance, allowing efficient s.c. absorption and prolonged circulation half‐life, two important attributes for further clinical development.

As mentioned above, the most common cause of death from cancer is metastatic spread [51]. In this regard, it is currently held that tumor‐associated proteases play important roles in the EMT and in extracellular matrix degradation [31, 52, 53, 54] that enable some of the most crucial steps in carcinogenesis, in general, and in cancer metastasis in particular. The complexity of these roles dictates the necessity to address multiple challenges in the development both of efficacious inhibitors targeting tumor‐associated proteases and of technologies for their site‐specific delivery; these include challenges pertaining to the renal clearance, rapid metabolism, off‐target effects, and stability of the putative inhibitors in the protease‐rich tumor environment. Many of these demands have indeed been mastered for both APPI‐3M and APPI‐4M [25, 44]. For example, our earlier study highlighted the importance of using methods that identify and isolate proteins with high proteolytic stabilities, strong target affinity, and specificity, thereby enabling long on‐target residence time [25, 44]. These characteristics facilitated the enhanced accumulation of both APPI proteins in tumors that express high levels of the target proteases. In the current study, we confirmed the cumulative effect of the inhibitors in three orthotopic models, and, importantly, we did not detect the accumulation of the proteins in cancer‐free tissues in any of the three models.

APPI‐4M accumulated only in primary ovarian tumors and not in metastatic sites. This finding is supported by published clinical data showing a high concentration of KLK6 at the early stages of ovarian cancer and low concentrations at later stages of cancer development [55]. KLK6 has thus become an emerging target in the field of ovarian cancer therapeutics, since it is present at the early stages of ovarian cancer progression [56, 57]. It has been suggested that KLK6 is responsible for increased E‐cadherin shedding, which increases metastasis and ascites in epithelial ovarian cancer [58, 59]. To the best of our knowledge, there are currently no articles describing attempts to inhibit this protease *in vivo*—other than this study of a KLK6 inhibitor in a preclinical cancer model. We exploited the fact that OVCAR‐3 cells express high levels of KLK6 to test our KLK6 inhibitor, APPI‐4M‐HSA, in the orthotopic xenograft OVCAR‐3 mouse model. The advantages of this model [48] are twofold: first, the direct grafting of a small piece of ovarian tumor tissue derived from a tumor xenograft onto the ovary of a female mouse serves to maintain the tumor in a physiological environment, and, second, the development of ascites and metastases in the peritoneal cavity, liver, and diaphragm mimics the progression of ovarian cancer in humans. In the current study, we further improved this model by using luciferase‐expressing OVCAR‐3 cells, which allowed us to follow primary tumor and metastasis progression throughout the course of the experiment. Importantly, the use of luciferase enabled the detection of small metastatic sites in the intestines, kidneys, and heart that could not be detected in the less sensitive original model. This luminescence‐based model could be used as a new standard orthotopic model for ovarian cancer.

The prognosis for women who develop ovarian cancer is directly related to the stage of disease at the time of diagnosis [60]. Those diagnosed at stage I have a 5‐year survival of 90%. If the disease has spread to adjacent tissues, 5‐year survival rates drop to around 80% and to 25% in women with metastatic disease. In our mouse model (in which the tumor was stitched into one ovary), APPI‐4M‐HSA showed promising results, significantly reducing the metastatic burden of OVCAR‐3 cells in ovaries, liver, kidneys, heart, and lungs, while maintaining a good safety profile. Furthermore, during the 30‐day treatment regimen, we did not observe side effects or treatment‐related fatalities. These results encourage further study of the role of KLK6 in ovarian cancer progression and the possibilities – and utility – of targeting it. In this context, APPI‐4M‐HSA could be a powerful player.

Cancer invasion patterns into skeletal muscle are guided by molecular and physical interphases within myocytes, as well as within blood vessels and nerves. Neoplastic invasion into skeletal muscle is associated with worsened cancer prognosis, as skeletal muscle invasion hinders body function, imposes a significant surgical challenge and compromises quality of life after radical resection [61]. In this study, histopathological assessment of the lungs of MDA‐MB‐231 breast tumor‐bearing mice, on both the macro and micro levels, indicated that the APPI‐3M‐HSA treatment not only reduced the metastatic burden but also contributed to local control of the disease. In the group treated with APPI‐3M‐HSA, surgery was easier, as the solid tumors were smoothly excised, while in the untreated group, the tumors were much more difficult to excise and histopathological specimens contained more adjacent striated muscle into which the tumors had invaded. Moreover, in the untreated group, the neoplastic cells had infiltrated intramuscularly within the interstitial endo‐ and perimysium, between and along muscle cells and fascicles in striated muscle tissue. The promising results obtained here for APPI‐3M‐HSA treatment indicate that the use of this agent could also be extended to its administration as a neoadjuvant prior to surgery; this notion, however, remains a subject for future studies. Another direction for future study would be an extensive investigation of the metabolomic expression of KLK6 and mesotrypsin in muscle tissues adjacent malignancies as a means to assess their function and possible role in invasion, but this direction, too, is beyond the scope of the present study.

The part of this study devoted to APPI‐3M‐HSA comprises the first report of the *in‐vivo* use of a mesotrypsin inhibitor. During the past decade, mesotrypsin has been associated with different stages of cancer development and with several types of malignancy, including lung, colon, breast, pancreas, and prostate cancer [9, 10]. A clue to the role played by mesotrypsin in metastasis may be found in its enhanced catalytic capability to hydrolyze canonical trypsin inhibitors [21]. These inhibitors include human Kunitz protease inhibitor domains from amyloid precursor‐like protein 2 (APLP2), bikunin, hepatocyte growth factor activator inhibitor type 2 (HAI2), and others [43, 62], all highly abundant in the TME. It has been suggested that the inhibitors act as physiological substrates for mesotrypsin, leading to their cleavage and inactivation, thereby contributing to the significant role of mesotrypsin in the mechanism of metastasis enhancement. It would, therefore, seem possible that delivering a potent and selective mesotrypsin inhibitor into the protease‐rich environment of a tumor would “protect” natural inhibitors of other proteases from cleavage and inactivation. As a result, homeostasis – and hence the imbalance in proteolytic enzyme activity – in the TME could be restored. Here, we show for the first time the potential of targeting mesotrypsin with a protein‐based inhibitor *in vivo*, whereas this potential has been investigated previously only in an *in‐vivo* prostate cancer model by genetically silencing mesotrypsin expression [9]. In our prostate and breast cancer orthotopic models, it is evident that introducing a mesotrypsin‐targeting inhibitor has an inhibitory effect on tumor growth and metastasis. We hypothesize that these phenotypes result from the “salvage” of natural inhibitors, as APPI‐3M‐HSA selectively binds to mesotrypsin and prevents it from inactivating mesotrypsin inhibitors and potentially also other inhibitors of pro‐metastatic proteases in the TME. This *in vivo* functional inhibitor of mesotrypsin could be used as a tool to support research on the mesotrypsin mechanism, as it provides exciting new opportunities for a better understanding of this target in different cancer models.

Recently, we showed that APPI‐4M could be leveraged as tool to investigate the role of KLK6 in the TME of pancreatic ductal adenocarcinoma [63] In that model, the selective and robust inhibition of KLK6 by APPI‐4M also reduced KLK6 mRNA expression, cell metabolic activity, and KLK6 secretion, but, in turn, increased the secretion of other serine and aspartic lysosomal proteases. As mesotrypsin and KLK6 are thought to be involved in different cancer stages and various types of cancer, we believe that APPI‐3M, APPI‐4M, and their HSA fusion proteins can be used in all phases of drug development, ranging from *in‐vitro* to *in‐vivo* studies, and in studies devoted to a better understanding of the mechanism of action of mesotrypsin and KLK6 and to dissecting their roles at the molecular level and in animal models for cancer and other malignancies.

## 5 Conclusion

This work describes the characterization and *in vivo* testing of novel anti‐metastatic serine protease inhibitors that can be used for cancer therapy and diagnostics. We focus on mesotrypsin and kallikrein 6 (KLK6) as protease targets and on prostate, breast, and ovarian cancer as proof‐of‐concept. However, as these inhibitors have an encouraging pharmacokinetic profile and a decisive pharmacodynamic phenotype, they can potentially be used for other serine proteases and other types of cancer.

## Conflict of interest

The authors declare no conflict of interest.

## Author contributions

AS and NP contributed to conceptualization and writing—original draft preparation; AS, IC, OB‐D, RAS, KMY, DS, ME, and NP contributed to methodology; AS, IC, IA, OB‐D, RAS, KMY, DS, ME, and NP contributed to validation, investigation, and writing—review & editing; AS, IC, IA, DS, ME, and NP contributed to formal analysis; NP contributed to resources, project administration, and funding acquisition; AS, IC, RAS, and KMY contributed to data curation; AS, IC, DS, ME, and NP contributed to visualization; ME and NP contributed to supervision. All authors provided critical feedback and helped shape the research, analysis, and manuscript.

### Peer review

The peer review history for this article is available at https://www.webofscience.com/api/gateway/wos/peer‐review/10.1002/1878‐0261.13513.

## Supporting information


**Fig. S1.** APPI‐3M treatment suppresses metastasis formation in the lung, liver, spleen, and digestive system.
**Fig. S2.** No major differences in total or organ weights were recorded upon treatment with APPI‐3M.
**Fig. S3.** APPI‐3M was either PEGylated with 20 kDa PEG or fused to human serum albumin (HSA).
**Fig. S4.** Slow tight‐binding inhibition of KLK6 catalytic activity by APPI variants.
**Fig. S5.** APPI‐3M‐HSA treatment reduced metastasis formation in an orthotopic breast cancer model.
**Fig. S6.** Histopathological examination supported the findings that the treatment with APPI‐3M‐HSA improved local control of disease.
**Table S1.** Systemic exposure to APPI‐3M, APPI‐4M, and the APPI‐3M‐HSA, and APPI‐4M‐HSA fusion proteins following i.v. and s.c. administration (mean, *n* = 4).

## Data Availability

Supporting data are available as Supporting Information.
