# Supplementary material for: Serine protease inhibitors decrease metastasis in prostate, breast, and ovarian cancers
Source: Mol Oncol. 2023 Sep 25;17(11):2337–55. doi: 10.1002/1878-0261.13513 (PMC10620120; doi:10.1002/1878-0261.13513)

**Supplementary information**

**Figure S1.** APPI-3M treatment suppresses metastasis formation in the lung, liver, spleen, and digestive system. The figure shows *ex vivo* bioluminescence of total body (A), lung (B), liver (C), spleen (D), digestive system (E), and primary tumor (F). Data for individual groups are shown as means ± SD (n=6). One-way ANOVA followed by Tukey’s multiple comparison test was used to determine the statistically significant differences between the groups (luminescence signal values shown above each treatment in the figure).

**Figure S2:** No major differences in total or organ weights were recorded upon treatment with APPI-3M. At the study end point, all mice were sacrificed, and selected organs were harvested and weighed. Organ weight is plotted as a percentage of total body weight at the study end point. Group mean values (exact values are given in the data set labels in the graphs) ± SEM are plotted vs. treatment. Data was statically analyzed using single factor one way ANOVA followed by Tukey’s multiple comparison test.

**Figure S3:** APPI-3M was either PEGylated with 20 kDa PEG or fused to human serum albumin (HSA). (**A)** PEGylation was performed using N-hydroxysuccinimide (NHS)-functionalized polyethylene glycol (PEG-NHS), which reacts with either the lysine residues or the free N-terminus of APPI. Since there are 3 lysine residues in the APPI-3M scaffold, an expected maximum of 4 labels correspond to four bands. Based on ImageJ software gel band intensity measurements of the material that was recovered after the PEGylation process, our results showed an overall 72% PEGylation yield from this material, with 55% of APPI labeled once, 12% labeled twice, 2.8% labeled 3 times, and 2.3% labeled 4 times (in four different positions). A large fraction of the APPI (28%) did not react with PEG. (**B)** Fusing APPI-3M to HSA produced a 77 kDa protein, as determined by Western blot using an anti-His antibody.

**Figure S4:** Slow tight-binding inhibition of KLK6 catalytic activity by APPI variants. The K_i_ of the reaction was calculated as described in the Materials and Methods section. V_0_ represents the uninhibited rate and V_i_ represents the rate in the presence of APPI. Experiments were performed in triplicate.


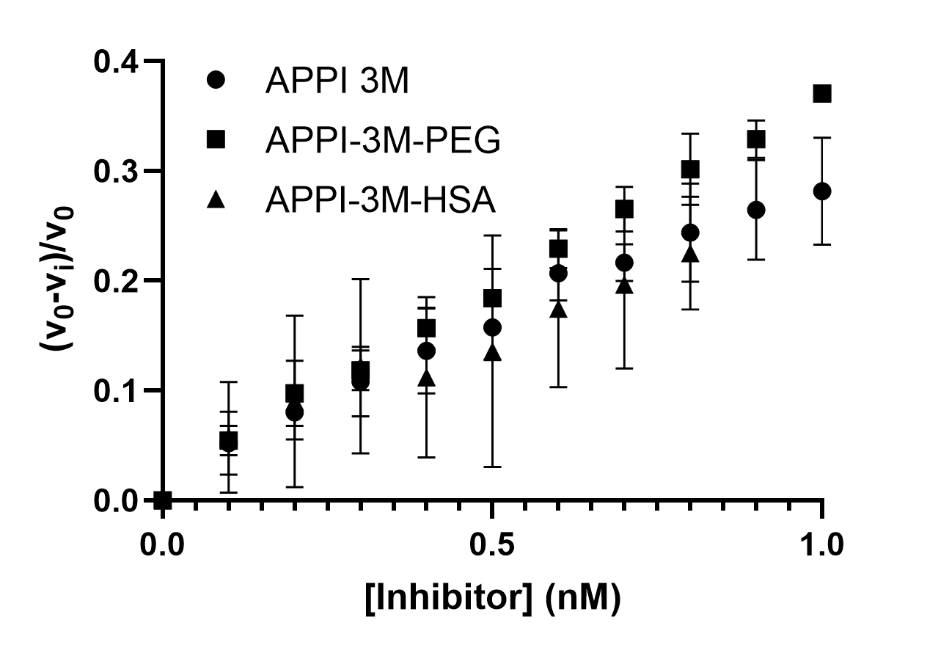


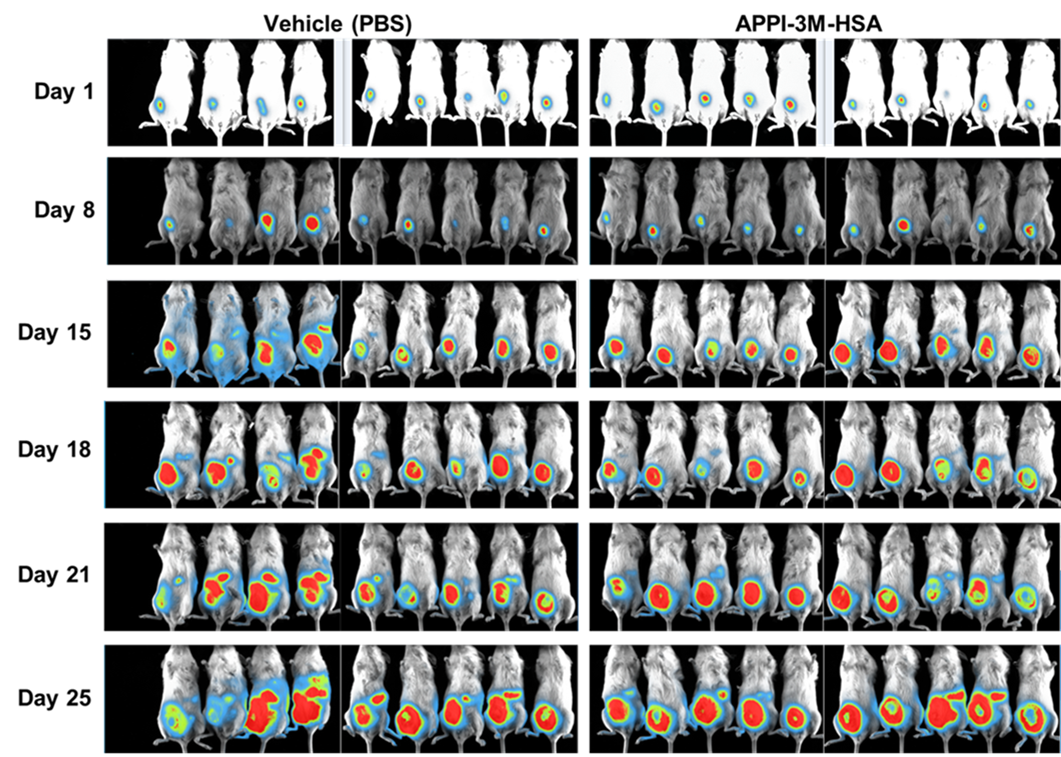


**Figure S5:** APPI-3M-HSA treatment reduced metastasis formation in an orthotopic breast cancer model. Mice were treated twice a week with s.c. injections of APPI-3M (82.5 mg/kg) or PBS for 25 days. n =10 in APPI-treated group; n = 9 in control group. .


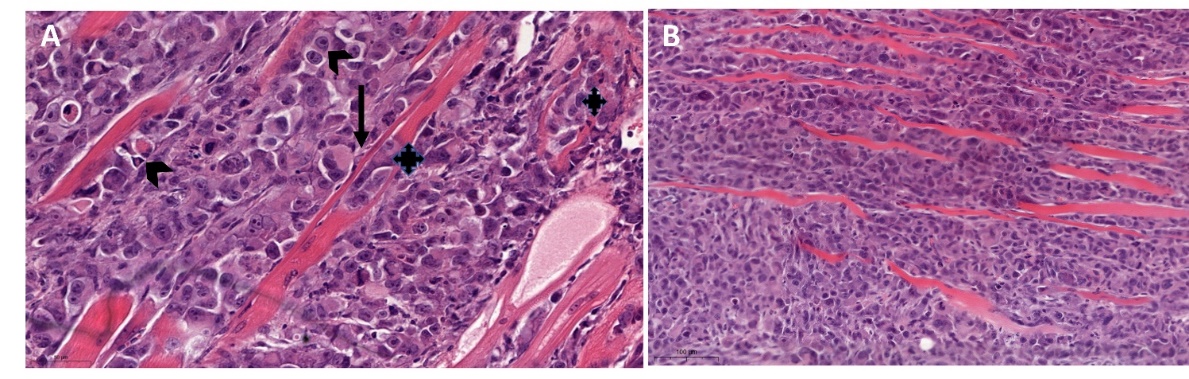

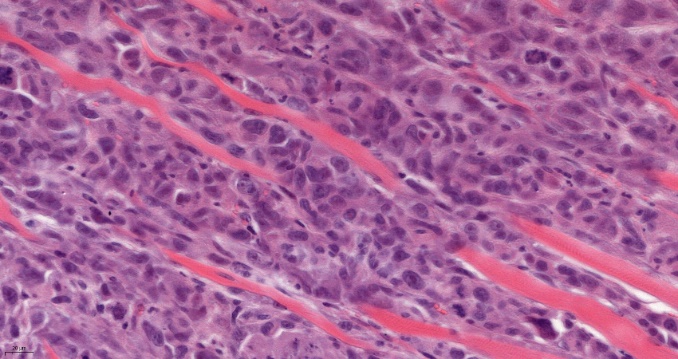


**B**

**Figure S6:** Histopathological examination supported the findings that the treatment with APPI-3M-HSA improved local control of disease. (A) In the untreated group, cancer cells infiltrated and invaded the striated muscle of adjacent organs (e.g., lungs). Muscle invasion occurred on both the longitudinal axis (black arrow) and the transversal axis (black arrowheads) alongside cystic-like degeneration (lower right). Neoplastic cells formed isolated cancerous islands within the muscle that gradually replaced the muscle fibers and destroyed them (asterisk). (B) In contrast, although the muscle fibers of the treated group were invaded by the adjacent carcinoma cells, there was no intra-fiber infiltration. ×40. Scale bar for panels A and B is 50 µm.

**Table S1:** Systemic exposure to APPI-3M, APPI-4M, and the APPI-3M-HSA, and APPI-4M-HSA fusion proteins following i.v. and s.c. administration (mean, n=4). The injected dose was equivalent to 30 µmol and 4 µmol of APPIs and APPI-HSAs, respectively. The terminal half-life and area under the curve (AUC) values were calculated using the non-compartmental analysis option of the PKSolver 2.0 Microsoft Excel add-on.


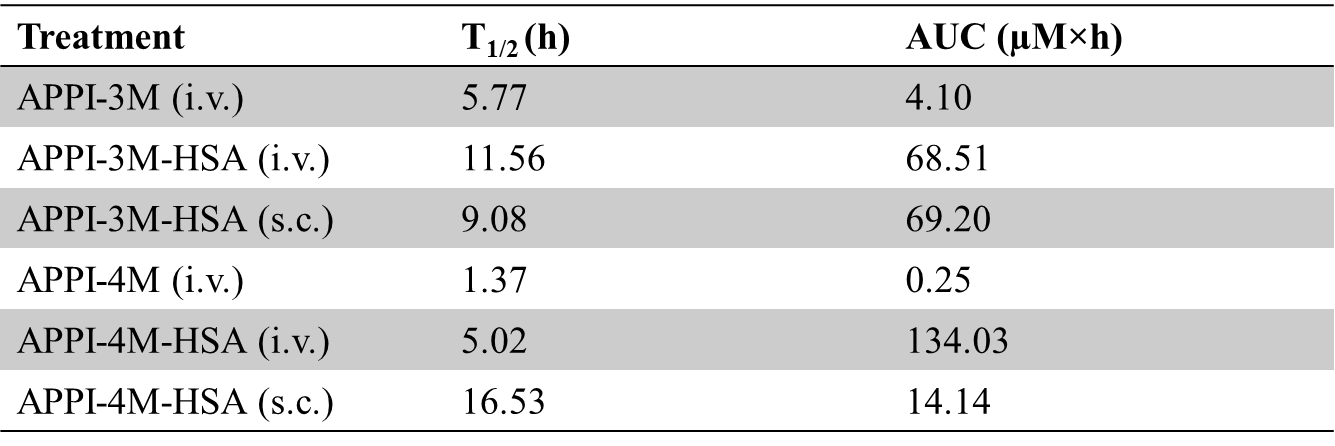

Supplement: Supplementary file 1 — Fig. S1. APPI‐3M treatment suppresses metastasis formation in the lung, liver, spleen, and digestive system. Fig. S2. No major differences in total or organ weights were recorded upon treatment with APPI‐3M. Fig. S3. APPI‐3M was either PEGylated with 20 kDa PEG or fused to human serum albumin (HSA). Fig. S4. Slow tight‐binding inhibition of KLK6 catalytic activity by APPI variants. Fig. S5. APPI‐3M‐HSA treatment reduced metastasis formation in an orthotopic breast cancer model. Fig. S6. Histopathological examination supported the findings that the treatment with APPI‐3M‐HSA improved local control of disease. Table S1. Systemic exposure to APPI‐3M, APPI‐4M, and the APPI‐3M‐HSA, and APPI‐4M‐HSA fusion proteins following i.v. and s.c. administration (mean, n = 4). [file MOL2-17-2337-s001.docx]
